# Accurate screening of patients with diabetes based on physical examination: pancreas imaging features from chest CT and laboratory data

**DOI:** 10.3389/fendo.2025.1698247

**Published:** 2025-11-26

**Authors:** Jixing Yi, Jiarun Lin, Qing Feng, Zhou Huang, Shu Li, Bumin Liang, Haohua Wu, Haiwei Yan, Eqing Yang, Jiaquan Wu, Yiqiong Liang, Xuanhan Liu, Jin Chen, Fengming Xu, Tao Li

**Affiliations:** 1 Department of Radiology, Liuzhou Worker’s Hospital, Liuzhou, Guangxi Zhuang Autonomous Region, China; 2 Medical Records Data Center, Liuzhou Worker’s Hospital, Liuzhou, Guangxi Zhuang Autonomous Region, China; 3 Department of Emergency, Liuzhou Worker’s Hospital, Liuzhou, Guangxi Zhuang Autonomous Region, China; 4 School of International Education, Guangxi Medical University, Nanning, Guangxi Zhuang Autonomous Region, China; 5 Information Department, Liuzhou Worker’s Hospital, Liuzhou, Guangxi Zhuang Autonomous Region, China; 6 Department of Radiology, Yangshuo County People’s Hospital, Guilin, Guangxi Zhuang Autonomous Region, China; 7 Department of Radiology, Guangxi International Zhuang Medicine Hospital, Nanning, China; 8 Department of Radiology, The Third Affiliated Hospital of Guangxi Medical University, Nanning, Guangxi Zhuang Autonomous Region, China; 9 Department of Radiology, Liuzhou Traditional Chinese Medical Hospital, Liuzhou, Guangxi Zhuang Autonomous Region, China

**Keywords:** CT, pancreas, diabetes mellitus, fasting blood glucose, diagnostic model

## Abstract

**Background:**

Fasting plasma glucose (FBG) was used in the large-scale primary screening of diabetes mellitus (DM). However, some people who actually have DM have normal fasting glucose (NFG), which shows a high false negative rate.

**Objective:**

To investigate the application value of pancreatic radiomics features and laboratory data from chest CT scan in screening DM patients.

**Methods:**

Patients with DM were diagnosed according to HbA1c≥48 mmol/mol(6.5%). The radiomics features of pancreas in lung window (L) and soft tissue window (S) of chest CT and laboratory data of 3587 patients from D1 (model training and testing) and D2/D3/D4/D5 (external validation) were retrospectively analyzed to construct a diagnostic model for DM screening.

**Results:**

The AUC of the lung window-laboratory models (L-Lab-LR and L-Lab-SVM) in the test set were 0.958/0.965. The AUC of soft tissue windows-clinical models (S-Lab-LR and S-Lab-SVM) were 0.958/0.969, 0.875/0.881, 0.935/0.959, 0.905/0.919, respectively.

**Conclusions:**

The diagnostic model based on chest CT pancreatic radiomics features and laboratory data has a very high diagnostic efficiency, which is accurate and reliable for the initial screening of DM patients. This method will facilitate early diagnosis of those individuals with DM who may have been missed.

## Highlights

1. Chest CT pancreatic radiomics features and laboratory data can accurately identify individuals with diabetes.
2. Chest CT pancreatic radiomics features and laboratory data can greatly reduce the false negative rate of fasting blood glucose for initial screening of diabetic patients.

## Introduction

Diabetes mellitus (DM) is a chronic ailment stemming from absolute or relative insufficiency in insulin secretion and utilization disorders, which is typified by hyperglycemia (1, 2). According to statistical data, as of 2021, approximately 537 million individuals worldwide were afflicted with DM. It is anticipated that the number of DM patients will rise to 643 million by 2030 and reach 783 million by 2045 (3). In the early phase of the disease, DM patients frequently exhibit non - significant clinical manifestations, resulting in frequent delays in DM diagnosis, which may give rise to severe complications and even life-threatening outcomes (4). The early detection and screening of DM hold significant implications for preventing the continuous deterioration of the disease. Simultaneously, the implementation of cost - effective DM diagnostic strategies is also the crux for ensuring the efficient utilization of medical resources. Currently, although fasting blood glucose (FBG), oral glucose tolerance test (OGTT), and glycosylated hemoglobin (HbA1c) are commonly employed indicators for the clinical diagnosis of DM, in routine large - scale DM screening, fasting blood glucose (FBG) remains the primary reliance (5). Nevertheless, certain studies have indicated that the fasting blood glucose (FBG) indicators employed for diabetes mellitus (DM) screening may significantly underestimate the actual prevalence of DM, exhibiting a high false negative rate (6). This implies that these DM patients might be overlooked owing to their seemingly normal FBG levels (FBG lower than the 6.1 mmol/L established by the World Health Organization (WHO) (7)), thereby elevating the risk of missed diagnosis. Therefore, a more reliable and accurate screening method is needed for the early screening of DM.

In the context of the exponential growth of data volume, machine learning has been extensively applied in the field of DM. A multitude of advanced computational methods have emerged to predict DM risk by integrating the essence of conventional statistical analysis or machine learning, and have demonstrated remarkable efficacy in DM risk stratification (8, 9). Particularly in developing countries, where the prevalence of DM has witnessed a substantial increase, the utilization of predictive models constructed through machine learning to identify individuals at high risk of DM for targeted further testing appears to hold the potential as an effective alternative to mass screening strategies. However, although the majority of prediction models focus on differentiating individuals with abnormal fasting glucose or pre - DM from normal individuals, these models encounter challenges in accurately identifying DM individuals with normal fasting glucose (NFG) within a large population, especially when fasting blood glucose (FBG) is employed as the criterion for DM screening (8, 9). Recently, a large - scale machine learning model based on the clinical data of over 60,000 individuals exhibited favorable diagnostic performance in the diagnosis of DM individuals with NFG (10).With the continuous advancement of medical imaging technology and radiomics, macroscopic image data can be deeply explored to reflect numerous high-level data that are imperceptible to the naked eye. Numerous studies have indicated that radiomics technology can be applied to the differential diagnosis of various diseases, the prediction of tumor metastasis, the prediction of benign and malignant tumors, the evaluation of treatment efficacy, and the prognosis of diseases after treatment, etc., and has demonstrated excellent predictive performance (10). In recent years, many studies have attempted to surmount the limitations of visual image assessment and elucidate the relationship between pancreatic radiomics features and DM (11, 12). Nevertheless, the two current studies evaluating the relationship between pancreatic radiomics and DM utilized abdominal localization (13). As is well-known, chest computed tomography (CT) has become an important means of routine disease screening in medical institutions, which is highly conducive to the opportunistic screening of large groups of asymptomatic patients (14–16). Compared with abdominal CT plain scan, chest CT plain scan is more frequently employed in the physical examination population and has become a common option in physical examination items. Therefore, this study aims to extract pancreatic radiomics features from routine chest CT examinations and combine them with laboratory data to construct an accurate diagnostic model that can ensure the efficient and comprehensive utilization of medical resources, thereby achieving the early and accurate screening of DM patients.

In this research, pancreatic radiomics features from chest CT and laboratory data were acquired from the plain chest CT scans of 3587 participants. Logistic regression (LR) and support vector machine (SVM) were employed for data analysis to construct distinct machine - learning diagnostic models, namely Lung window-laboratory-LR/SVM (L-Lab-LR/SVM) and soft tissue window-laboratory- LR/SVM (S-Lab-LR/SVM), with the aim of realizing the early and accurate screening of diabetes mellitus (DM) patients during routine physical examinations. The findings indicated that the trained models demonstrated satisfactory performance on the test set and multiple external validation sets (excluding the central D2 dataset). Moreover, in comparison with the screening of DM patients using only FBG, the false - negative rate was significantly reduced. Relying on routine physical examinations, the early detection and early diagnosis of DM can be accomplished.

## Materials and methods

### Research materials

**Figure 1:**
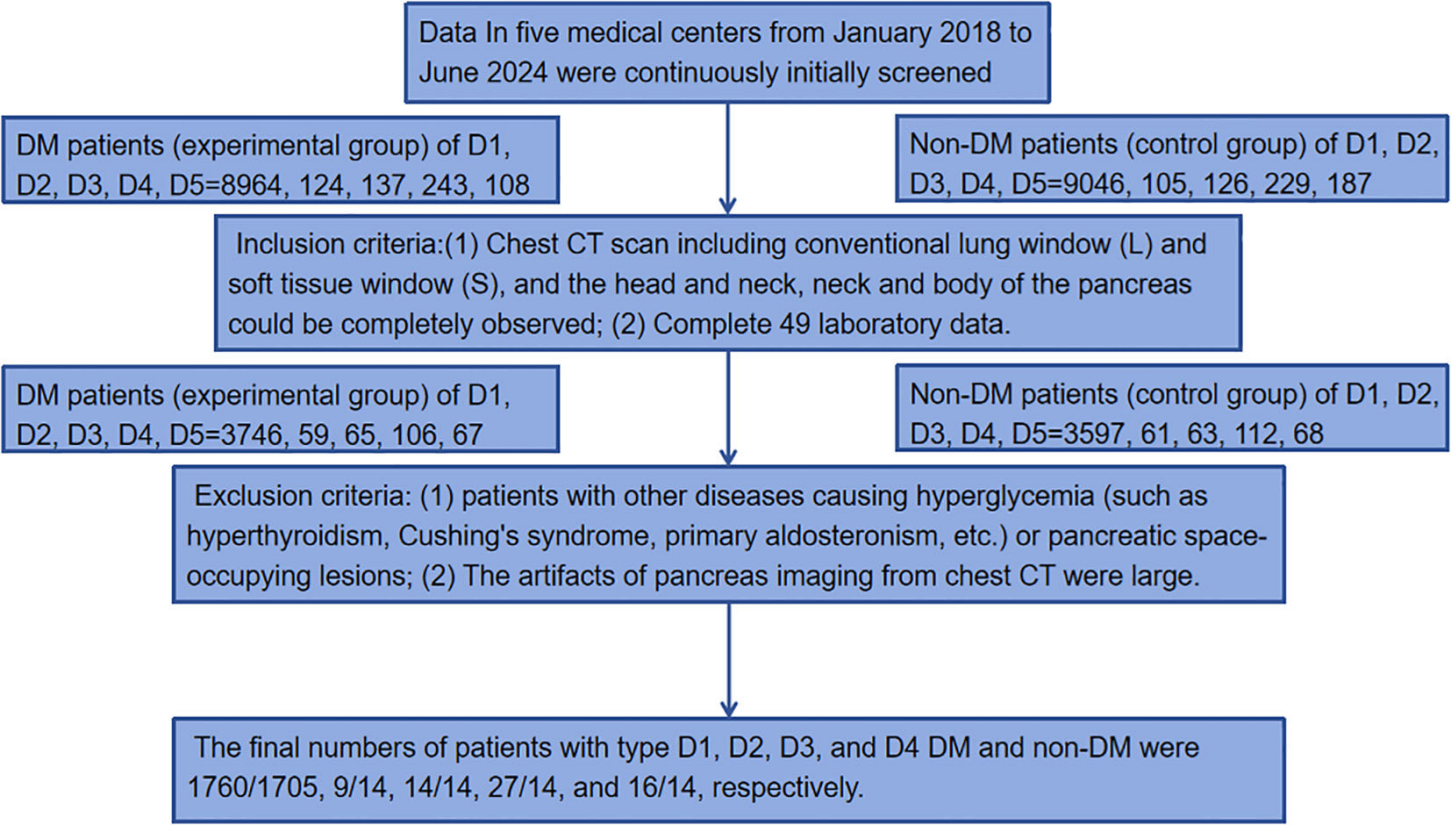
Flowchart of data screening.

The data were collected from five medical centers: Liuzhou Workers’ Hospital, Yangshuo County People’s Hospital, Guangxi International Zhuang Medicine Hospital, Liuzhou Traditional Chinese Medicine Hospital and Nanning Second People’s Hospital. The datasets were named D1, D2, D3, D4, and D5. DM was diagnosed according to HbA1c≥48 mmol/mol(6.5%) (10). Data of 9576(8964/124/137/243/108) DM patients (experimental group) and 9693(9046/105/126/229/187) non-DM patients (control group) in five medical centers from January 2018 to June 2024 were continuously initially screened. Inclusion criteria: (1) Chest CT scan including conventional lung window (L) and soft tissue window (S), and the head and neck, neck and body of the pancreas could be completely observed; (2) Complete 49 laboratory data: Hemoglobin_A1C(HA1C), Fasting_blood_glucose(FBG), Urea, creatinine, Purine_trione, Cystatin_C, CrCl, Microglobulin, Total_carbon_dioxide, Total_protein, Albumin, Globin, White_ball_than, Total_bilirubin, Bilirubin_direct, Indirect_bilirubin, AAT, Alkaline_phosphatase, Asparpartate aminotransferase, AST/ALT, R_glutamyl_transferase, TBA, Lactate_dehydrogenase, Cholinesterase, Prealbumin, Adenosine_deaminase, Fibronectin, Leucocyte_count, Neutrophile granulocyte1, Monocytes1, Lymphocytes, Acid-inducing_cells, Alkaloid_cells, Neutrophil(%), Lymphocytes(%), Monocytes(%), Acid-inducing(%), Alkaloid(%), RBC, hematocrit, Mean_RBC_volume, Mean_HGB_content, Mean_HGB_concentration, RBC distribution width(SD), RBC distribution width(CV), Platelet_count, PLT_distribution, Mean_PLT_volume, Large_platelet_ratio, Thrombocytocrit. Exclusion criteria: (1) patients with other diseases causing hyperglycemia (such as hyperthyroidism, Cushing’s syndrome, primary aldosteronism, etc.) or pancreatic space-occupying lesions; (2) The artifacts of pancreas imaging from chest CT were large. The final numbers of patients with type D1, D2, D3, and D4 diabetes mellitus (DM) and normal controls were 1760/1705, 9/14, 14/14, 27/14, and 16/14, respectively. The inclusion and exclusion process is roughly shown in **Figure 1**.

### CT scanning methods

Five kinds of MRI scanners were used to perform chest CT scans in five medical centers (Liuzhou Workers’ Hospital, Yangshuo County People’s Hospital, Guangxi International Zhuang Medicine Hospital, Liuzhou Traditional Chinese Medicine Hospital and Nanning Second People’s Hospital) respectively: GE64 slices CT scanner (General Electric), GE16 slices CT scanner (Lightspeed 16, GE Healthcare), Precision 128 Max (Campo Imaging), GE64 gemstone spectral CT (GE Discovery CT750HD, GE Discovery CT750HD, Campo Imaging) GE Healthcare) and GE 128-row Revolution CT ES (Revolution CT ES, GE Healthcare) (**Table 1**).

**Table 1:** Related scanning parameters of different scanners.

Scanning parameters	Medical center A	Medical center B	Medical center C	Medical center D	Medical center E
Tube voltage (kV)	120	120	120	100	100
Tube current (mA)	auto	180	50	auto	auto
FOV (mm2)	358×358	380×380	380×380	320×320	370×370
Slice thickness (mm)	5	5	5	5	5
Layer spacing (mm)	5	5	1.5	5	5
Collimator width (mm)	40	20	80	40	40
speed (s/r)	0.6	0.8	0.5	0.7	0.5
Pitch	0.984	1.75	1.01	1.531	0.992
Matrix	512×512	512×512	768×768	512×512	512×512

### Data processing and model construction

Pancreas imaging features from chest CT and laboratory data were uploaded to Darwin Research platform in DICOM format and CSV form, respectively (http://10.100.118.3:8089, data was only transmissed in hospital network, no data leak risk), which is launched by Yizhun Medical AI technology limited company, which is an artificial intelligence research platform for medical imaging (Refer to https://arxiv.org/pdf/2009.00908v1).The image-based feature extraction, machine learning model development, and statistical data analysis was carried out on Darwin Research platform.

### Data processing

**Figure 2:**
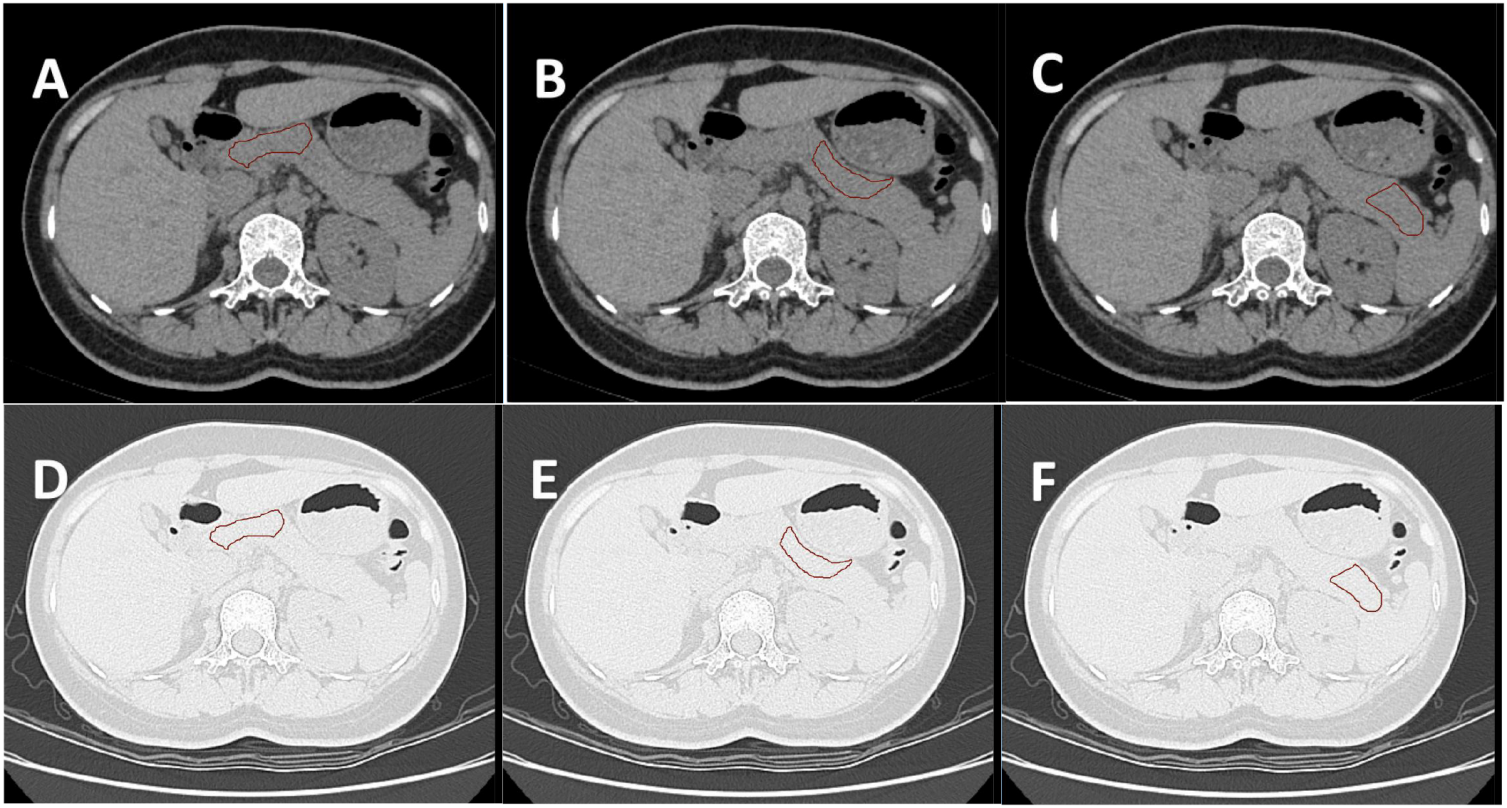
**(A–C)** show the ROI of the pancreatic head-neck, neck-body, and body-tail delineated by the soft tissue window of the same subject, respectively. **(D–F)** are the ROI of the lung window registered by the automatic registration function of the software, respectively. A total of six samples were obtained for one subject.

Image data processing specifically included the following steps: (1) Two-dimensional segmentation of Region of interest (ROI): Six samples of pancreatic head-neck, pancreatic neck-body, and pancreatic body-tail were delineated in the lung window and soft tissue window, respectively (12, 13) (**Figure 2**). The six obtained samples were respectively used as independent samples for feature extraction.

The intraclass correlation coefficient (ICC) was used to evaluate the intra-observer and inter-observer consistency of image feature extraction. The chest CT soft tissue window images of 20 positive patients were randomly selected from the training set for ROI segmentation and feature extraction. ROI segmentation was performed independently by two experienced radiologists (both with at least 5 years of chest and abdominal CT diagnosis experience and proficient in the Darwin Research platform). Four 2D features were randomly selected, and the intra-observer ICC was calculated by comparing the features extracted by observer A twice. The inter-observer ICC was calculated by comparing the features extracted by observer B with those extracted by the first observer A. When ICC>0.75 and P<0.05, the consistency was considered good. Under conditions with good agreement, the image segmentation task was averaged and randomly assigned to observers A and B.

(2) Feature extraction mainly includes shape features, first-order features, texture features and so on about 1125 image features which were contained in Darwin Research platform. In particular, the shape feature describes the basic geometric properties of the outlined area, such as size, shape and surface roughness; The first-order features use common basic metrics to describe the distribution of voxel intensity in the delineated region. The texture features include gray level co-occurrence matrix (GLCM), gray level free length matrix (GLRLM), gray level size area matrix (GLSZM), adjacent gray level color difference matrix (NGTDM) and gray level dependence matrix (GLDM). These features are designed to capture the spatial dependence of voxels in an image (17). The spatial heterogeneity characteristics of the image such as gray level change, spacer size and roughness were revealed. In addition, six kinds of filters including exponential, square, square root, log, log-sigma-3-0-mm-3D and wavelet are applied to further process the first-order and texture features. In particular, the wavelet filter extracts the key features from eight wavelet decomposed images. Finally, 1125 features were extracted from each sequence. The image data of CT scans from different centers were tuned through the ComBat method homogenization program based on the empirical Bayesian model correction method in the Darwin Research platform.

(3) Radiomics feature selection: Firstly, formula ① was used to standardize the features of each dimension of all samples to form data with a mean of 0 and a variance of 1.

(1) $$Z=\frac{X-mean}{\sqrt{var}}$$

*X* is the original data, mean is the mean of the original data, *var* is the variance of the original data.

Then, standardized data were normalized using the Minimum-maximum normalization(Formula ②) to eliminate the magnitudes of different features by scaling values to 0~1.

(2) $$Z=\frac{X-min}{max-min}$$

*X* is the original data, *min* and *max* are the minimum and maximum values of the original data, respectively.

The f_classif with its default parameters was used to screen out 30 valuable features for classification from 1125 image features. Least absolute shrinkage and selection operator(LASSO) and Logistic Regression(LR) were used to further screen out the most important imaging features. LASSO iteratively selected the relevant features according to the best parameter (alpha). In the K-fold cross validation performed by LR, the K value was 10, the evaluation index was roc_auc, and the penalty term and feature importance threshold were not set. Predict_score was constructed based on the selected optimal radiomics features (Refer to https://arxiv.org/pdf/2009.00908v1).

Selection of clinical features: 49 laboratory data were also standardized and normalized using formula ① and ② successively. Then f_classif was used to screen out 10 valuable features for classification. LASSO and LR were used to further screen out the most important clinical features. Similarly, LASSO iteratively selects the relevant features based on the best parameter (alpha). In the K-fold cross validation performed by LR, the K value was 10, the evaluation index was roc_auc, and the penalty term and feature importance threshold were not set.

### Model building and statistical method

The D1 dataset was divided into training set and test set according to 5:5. The D2,D3,D4 and D5 datasets were used for external tests. Logistic Regression (LR), as a generalized linear regression analysis model, is often used in fields such as data mining, automatic disease diagnosis, and economic forecasting. The LR model form has w ‘x +b, where w and b are the parameters to be found. logistic regression corresponds w ‘x +b to an implicit state p through the function L, p =L(w ‘x +b), and then the value of the dependent variable is determined based on the size of p and 1-p. LR estimates the occurrence probability of events based on the given independent variable dataset. Since the result is a probability, the range of the dependent variable is between 0 and 1, which satisfies the binary outcome of this study where the negative value DM is 1 and the non-dm is 0 (18).

Support Vector Machine(SVM) is a type of generalized linear classifier that performs binary classification of data in a supervised learning manner. Its decision boundary is the maximum margin hyperplane for solving the learning samples. SVM uses hinge loss to calculate empirical risk and adds a regularization term in the solution system to optimize structural risk. It is a classifier with sparsity and robustness. SVM conducts nonlinear classification through the kernel method and is one of the common kernel learning methods, which also satisfies the binary outcome of this study where the negative value DM is 1 and the non-dm is 0 (19).

The models were constructed using the LR/SVM model construction section of the Darwin research platform(Refer to https://arxiv.org/pdf/2009.00908v1). In the construction of the LR model in this study: the elasticnet is selected as the penalty parameters, with the penalty coefficient C = 1 and the l1_ratio=0.5. In the construction of the SVM model in this study: the kernel type is selected as rbf. Gamma is set to 1/n_features(configured as “auto”), and coef=0 for polynomial cores and sigmoid cores. The penalty coefficient C = 1.

LR and SVM were used to construct the diagnostic model based on the predict_score constructed by the optimal radiomics features and the optimal laboratory data: four different diagnostic models were used, including lung window-laboratory-logistic regression nomogram (L-Lab-LR), lung window-laboratory-support vector machine (L-Lab-SVM), soft tissue window-laboratory-logistic regression nomogram (S-Lab-LR) and soft tissue window-laboratory-support vector machine (S-Lab-SVM). The optimal penalty coefficient C was found in k-fold cross validation (K value =10), and the number of penalty coefficients C was uniformly sampled at log scale[1, 1e6] by default. ROC, AUC, Accuracy, Sensitivity and Specificity were used to comprehensively evaluate the diagnostic efficacy of the model.

**Figure 3:**
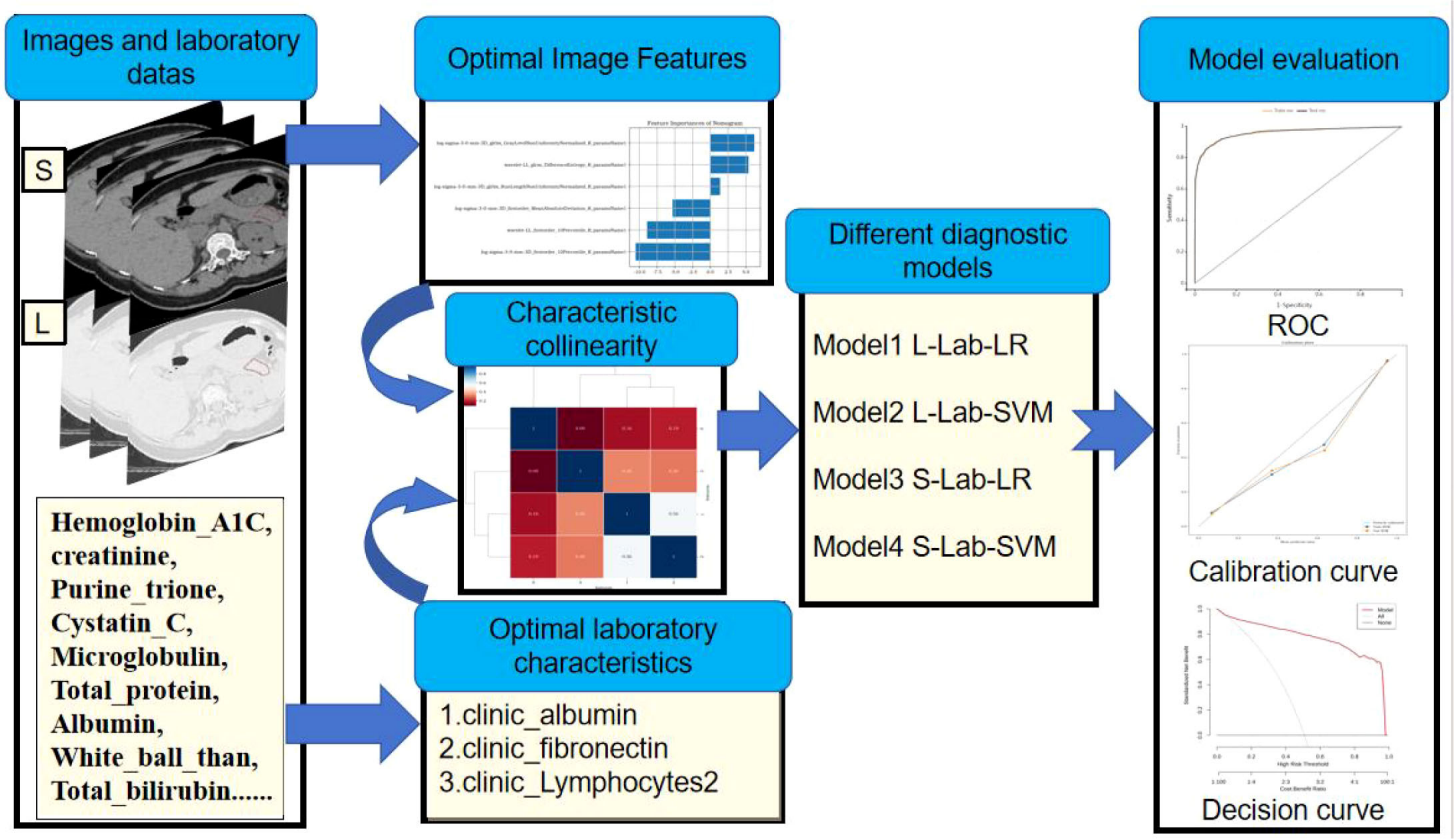
Simple process of data analysis and model construction: First, extract the image features from the CT image. Then, screen out the optimal model features. Build the model based on the selected optimal features. Finally, evaluate the diagnostic efficacy of the model.

Receiver operating curve (ROC) refers to the line connecting each point plotted under specific stimulus conditions, with the false alarm probability P (y/N) obtained by the subject under different judgment criteria as the abscissa and the hit probability P (y/SN) as the ordinate. ROC can help understand the balance between the sensitivity and specificity of classifiers. The Area under curve (AUC) is defined as the area enclosed by the ROC curve and the coordinate axes. Since the ROC curve is generally above the straight line y=x, the range of AUC values is between 0.5 and 1. The closer the AUC is to 1.0, the higher the authenticity of the detection method. When it equals 0.5, its authenticity is the lowest and it has no application value (20). The simple flow of data analysis and model construction is shown in **Figure 3**.

## Results

### Optimal radiomics features and laboratory clinical data

**Figure 4:**
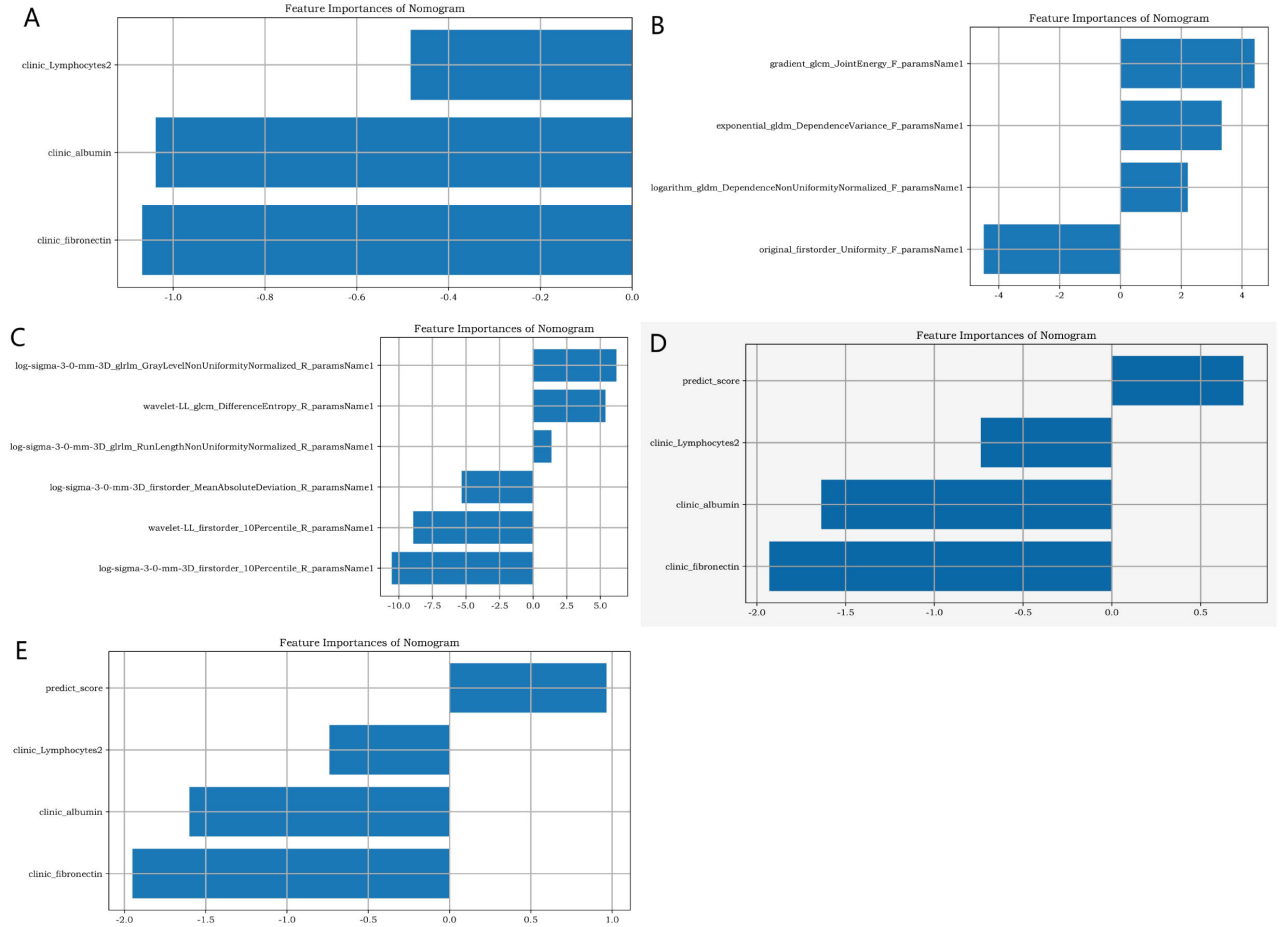
The importance map of the best radiomics features. **(A)** shows the best clinical feature from the clinic laboratory. **(B, C)** respectively display the optimal radiomics features of the pancreas in the lung window and soft tissue window of chest CT. **(D, E)** respectively present the joint radiomics features of the pancreas in the lung window and soft tissue window of chest CT.

D1, D2, D3, D4, and D5 encompassed 20,790, 138, 168, 246, and 180 image data samples (some of which were not identified by the software during the analysis). **Figure 4** presents the importance map of the optimally selected radiomics features for different sequences in the D1 group. The most outstanding laboratory data were clinic_albumin, clinic_fibronectin, and clinic_Lymphocytes2, respectively. **Table 2** presents the basic data and the most outstanding laboratory data of the DM group and non-DM group in D1 Center. The median age of the experimental group was 64 years (with an interquartile range of 56–72 years). There were 986 males (approximately 56.02%). The median age of the control group was 50.5 years (with an interquartile range of 42–56 years). There were 1067 males (about 62.58%).

**Table 2:** The demographics in D1 center.

Factor	Mean/ratio	Standard/ratio	Inter-quartile range (P25%-P75%)	Statistically significant (p value)
DM Age non-DM	64	13.18	56-72	p<0.001
	50.5	11.03	42-56	
DM Sex (males) non-DM	N=986 (56.02%)		/	p<0.001
	N=1067 (62.58%)		/	
DM HbA1c non-DM	10.41	2.73	8.10-12.40	p<0.001
	5.64	0.73	5.40-5.90
DM FBG non-DM	8.72	4.94	6.26-9.34	p<0.001
	5.17	1.14	4.74-5.35
DM OGTT non-DM	12.84	4.23	9.92-15.64	p<0.001
	7.22	1.58	5.78-8.48
DM Albumin non-DM	40.87	4.80	38.5-44.6	p<0.001
	45.18	2.98	43.90 -46.80
DM Fibronectin non-DM	318.03	105.99	256-366	p<0.001
	437.72	96.15	389-459
DM Lymphocytes2 non-DM	26.60	9.70	20.1-33.3	p<0.001
	32.65	7.97	27.7-37.8

Inter-observer and intra-observer reproducibility of Radiomics feature extraction. Four 2D features of the chest CT soft tissue window were randomly selected as follows:

MaximumDiameter_F_paramsName1, original_shape2D_MeshSurface_F_paramsName1, MinorAxisLength_F_paramsName1, Perimeter_F_paramsName1. The intraobserver ICC calculated based on the two measurements of observer A were: 0.796(95%CI=0.659-0.878), 0.797(95%CI=0.660-0.879), 0.927(95%CI=0.878-0.957), 0.901(95%CI=0.835-0.941), and all P <0.001. The inter-observer ICC calculated based on the measurements of two observers were: 0.812 (95%CI=0.686-0.888), 0.798 (95%CI= 0.662-0.879), 0.889(95%CI=0.815-0.934), 0.855(95%CI=0.757-0.913), and all P <0.001. This indicates that the intra-observer and inter-observer feature extraction has good consistency.

### Joint models construction and efficacy evaluation

**Figure 5:**
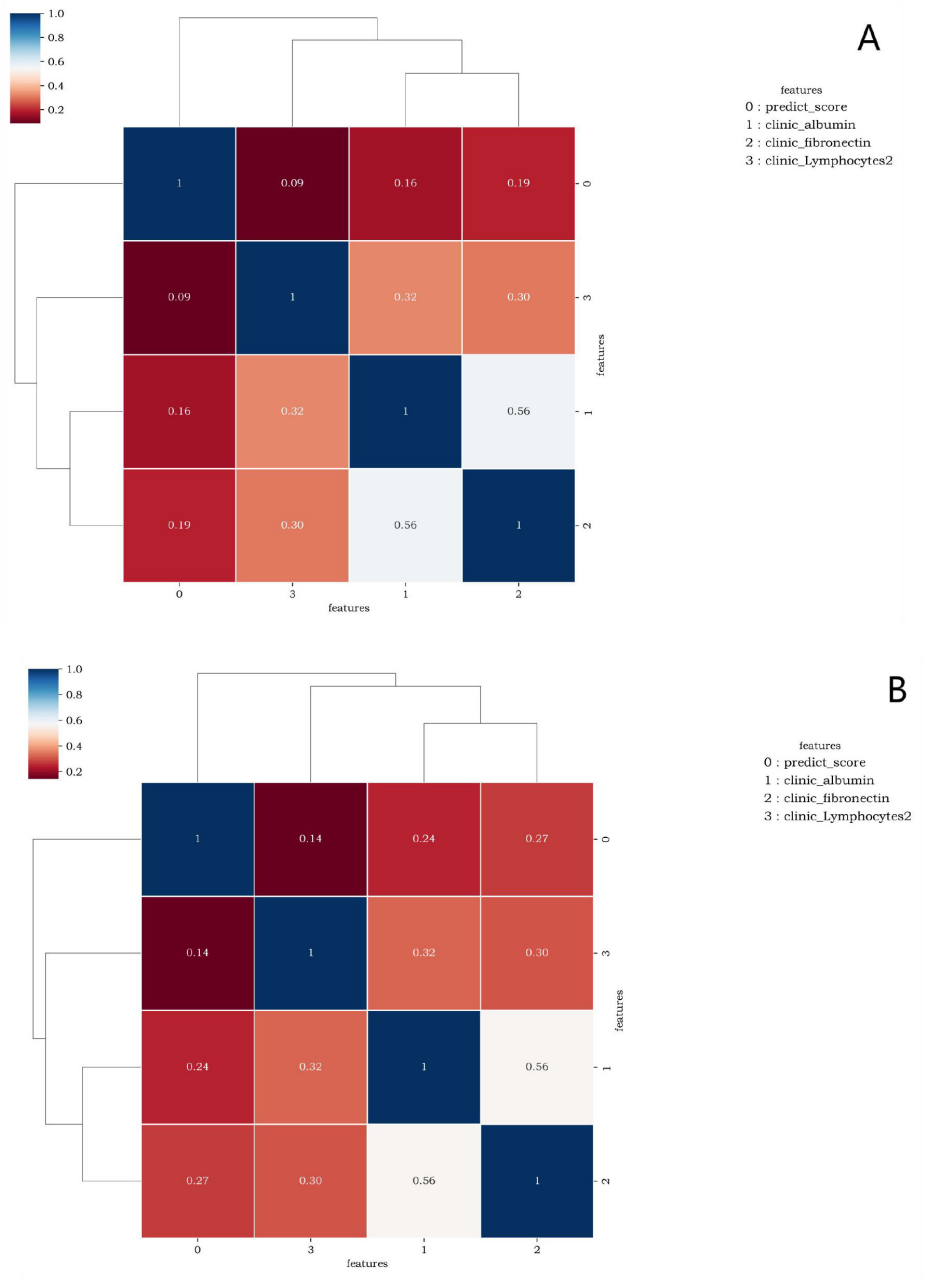
**(A, B)** are correlation graphs of predict_score and optimal laboratory features of the lung window and the soft tissue window, respectively. The correlation between different features ranged from 0.09 to 0.56 and from 0.14 to 0.56. This indicates that there is no collinearity or only weak collinearity between the features.

**Figure 6:**
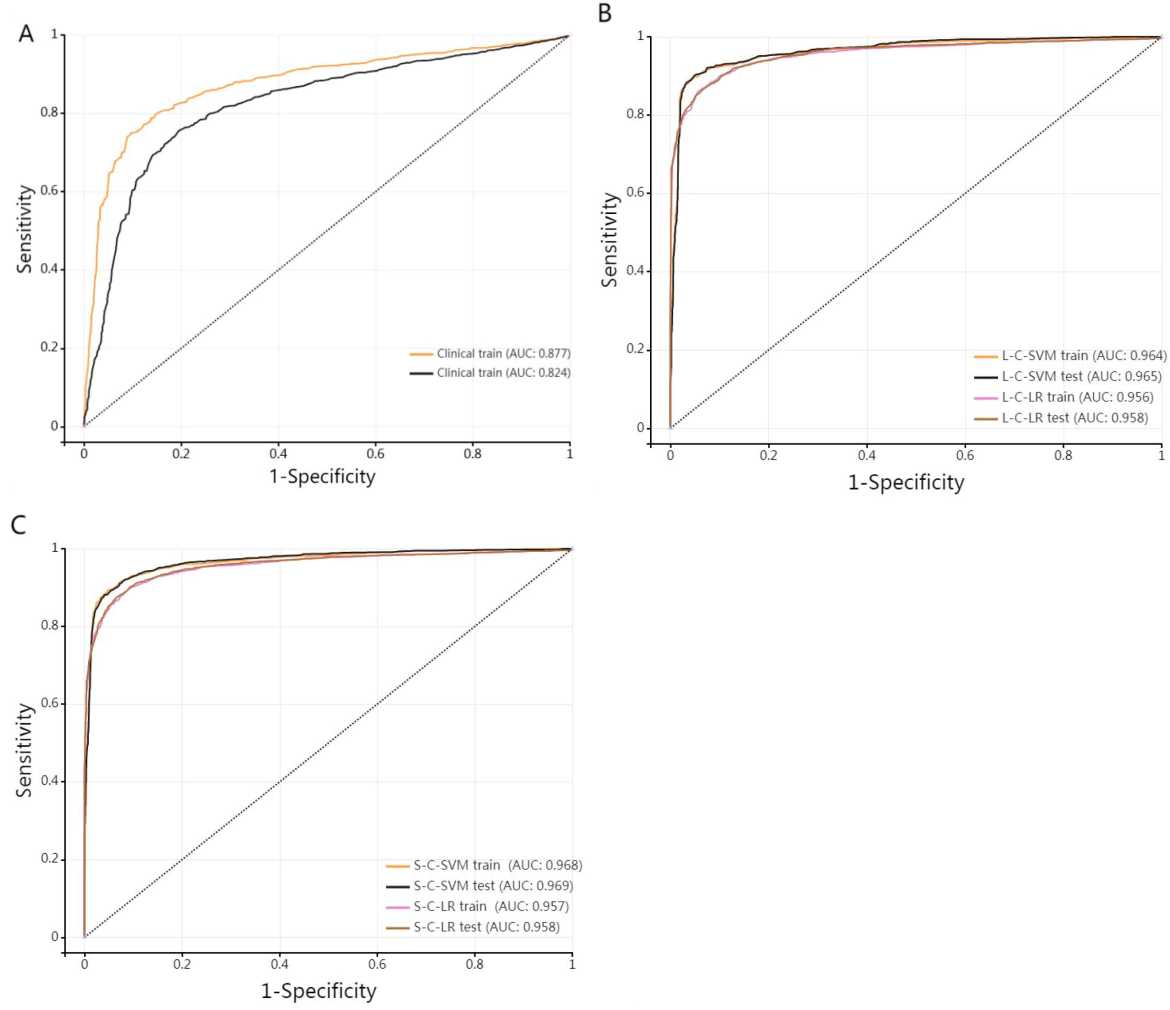
**(A)** stands for single-laboratory clinical model. **(B, C)** are ROC curves of the training set and test set of the four joint diagnostic models (L-Lab-LR with clinic, L-Lab-SVM with clinic, S-Lab-LR with clinic, and S-Lab-SVM with clinic) trained by D1(medical A), respectively.

**Figure 5** elucidates the collinearity between predict_score and laboratory data of the two sequences. The correlation coefficients among features are all<0.6, suggesting that there is no collinearity or weak collinearity among features. The ROC curves of the diagnostic models trained through the D1 group are presented in **Figure 6**. The results of AUC, accuracy, sensitivity, and specificity of the diagnostic model in group D1 are shown in **Table 3**. It is evident that while the single-laboratory clinical model demonstrated good diagnostic efficacy (AUC = 0.877), more ideal outcomes were achieved by constructing joint models that incorporated different imaging data.

**Table 3:** Predictive efficacy of different models.

Model	AUC (95%CI)	Sensitivity (95%CI)	Specificity (95%CI)	Accuracy	Positive predictive value (95%CI)	Negative predictive value (95%CI)
Lab_train	0.877 (0.867, 0.887)	0.75 (0.734, 0.766)	0.903 (0.89, 0.914)	0.82	0.901 (0.888, 0.913)	0.753 (0.736, 0.768)
Lab_test	0.824 (0.812, 0.836)	0.754 (0.738, 0.77)	0.807 (0.79, 0.822)	0.778	0.829 (0.814, 0.843)	0.726 (0.708,0.743)
L-Lab-LR_train	0.956 (0.951, 0.962)	0.862 (0.849, 0.875)	0.943 (0.933, 0.951)	0.902	0.939 (0.929, 0.948)	0.869 (0.856, 0.881)
L-Lab-LR_test	0.958 (0.953, 0.964)	0.872 (0.859, 0.884)	0.928 (0.917, 0.937)	0.899	0.926 (0.914, 0.935)	0.875 (0.862, 0.887)
L-Lab-SVM_train	0.964 (0.959, 0.969)	0.891 (0.879, 0.902)	0.96 (0.952, 0.967)	0.925	0.958 (0.95, 0.966)	0.895 (0.883, 0.906)
L-Lab-SVM_test	0.965 (0.96, 0.97)	0.901 (0.889, 0.912)	0.951 (0.942, 0.959)	0.926	0.95 (0.941, 0.958)	0.903 (0.891, 0.914)
S-Lab-LR_train	0.957 (0.951, 0.962)	0.9 (0.888, 0.911)	0.907 (0.895, 0.918)	0.903	0.909 (0.897, 0.919)	0.898 (0.886, 0.909)
S-Lab-LR_test	0.958 (0.953, 0.964)	0.875 (0.862, 0.887)	0.935 (0.925, 0.944)	0.905	0.933 (0.922, 0.942)	0.878 (0.866, 0.89)
S-Lab-SVM_train	0.968 (0.963, 0.972)	0.891 (0.878, 0.902)	0.954 (0.945, 0.961)	0.922	0.952 (0.943, 0.96)	0.894 (0.882, 0.905)
S-Lab-SVM_test	0.969 (0.964, 0.973)	0.881 (0.868, 0.893)	0.959 (0.951, 0.966)	0.919	0.957 (0.948, 0.964)	0.886 (0.874, 0.897)

Different training of joint models demonstrated very high AUC values (all > 0.95), indicating that the four training models have excellent diagnostic efficacy in diagnosing DM. The higher the sensitivity, the stronger the diagnostic method’s ability to identify patients, which can reduce the possibility of missed diagnosis. The higher the specificity, the stronger the diagnostic method’s ability to exclude non-patients, which can reduce the possibility of misdiagnosis. The specificity and sensitivity of single-laboratory clinical model achieved higher performance after combining the image data. The S-Lab-LR training model had the highest sensitivity of 0.9(0.888, 0.911), and the L-Lab-SVM training model had the highest specificity of 0.96(0.952, 0.967). It is indicated that the S-Lab-LR training model is better in identifying DM patients. If it is to reduce misdiagnosis, the L-Lab-SVM training model is better. The DeLong test was used to compare the AUC of different diagnostic models. The AUC of L-Lab-SVM_train is significantly better than that of L-Lab-LR_train (0.964 vs 0.956, Z = 3.113, p < 0.001). The AUC of S-Lab-SVM_train is significantly better than that of S-Lab-LR_train (0.969 vs 0.957, Z = 4.835, p < 0.001). The AUC of S-Lab-SVM_train is significantly better than that of L-Lab-SVM_train (0.969 vs 0.964, Z = 5.375, p < 0.001). It can roughly be considered that S-Lab-SVM has the most significant AUC value.

B and C show that the AUC of the joint model constructed by combining clinical laboratory data with imaging data has been significantly improved (the AUC of both the training group and the test group of the joint model is greater than 0.95).

**Figure 7:**
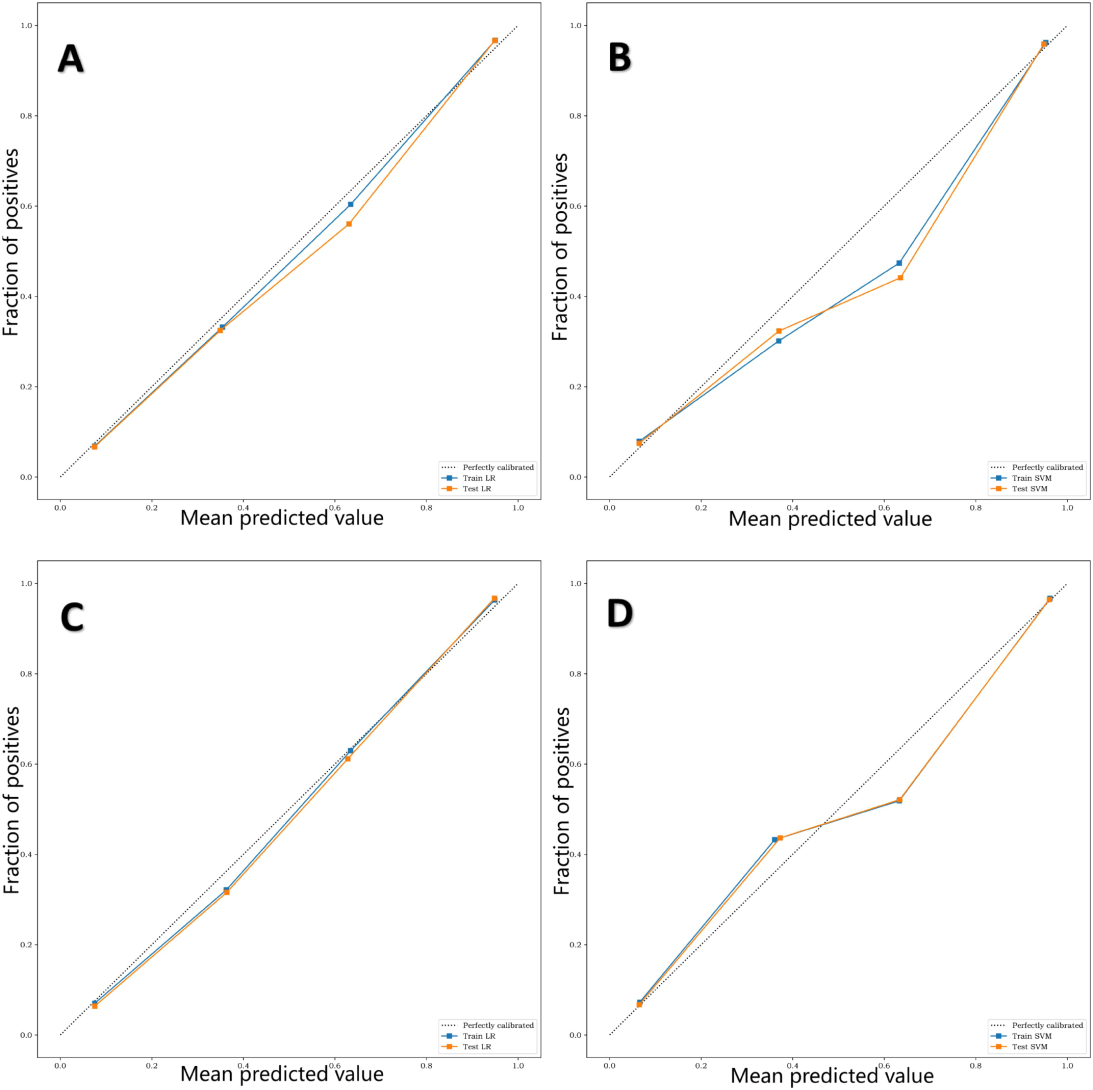
**(A–D)** are calibration curves of training sets and test sets of four different diagnostic models, L-Lab-LR, L-Lab-SVM, S-Lab-LR and S-Lab-SVM, respectively. The closer the fit of the calibration curve of the diagnostic model to the true curve (dashed line), the better the prediction performance. It can be seen that the L-Lab-LR and S-Lab-LR models have better predictive performance, while the L-Lab-SVM and S-Lab-SVM models have slightly poorer predictive performance.

**Figure 8:**
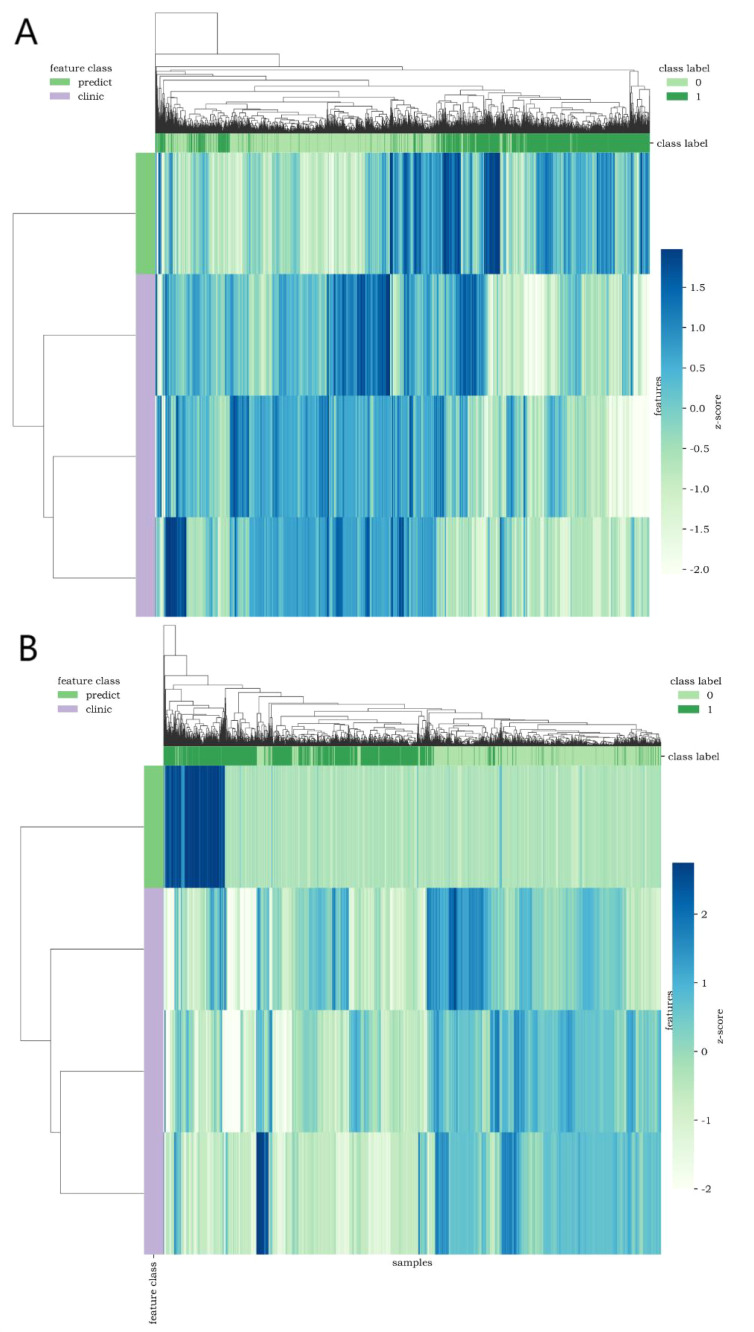
Cluster heatmap [**(A, B)** show CT lung window-laboratory characteristics and CT soft tissue window-laboratory characteristics respectively] uses different colors to reflect the value of each one-dimensional feature (row) of the sample (column). The top color bar expresses the true class of the samples, the left color bar expresses the class of the features, and the dendrogram shows the results of the hierarchical clustering. (1=DM, 0=non-DM).

**Figure 9:**
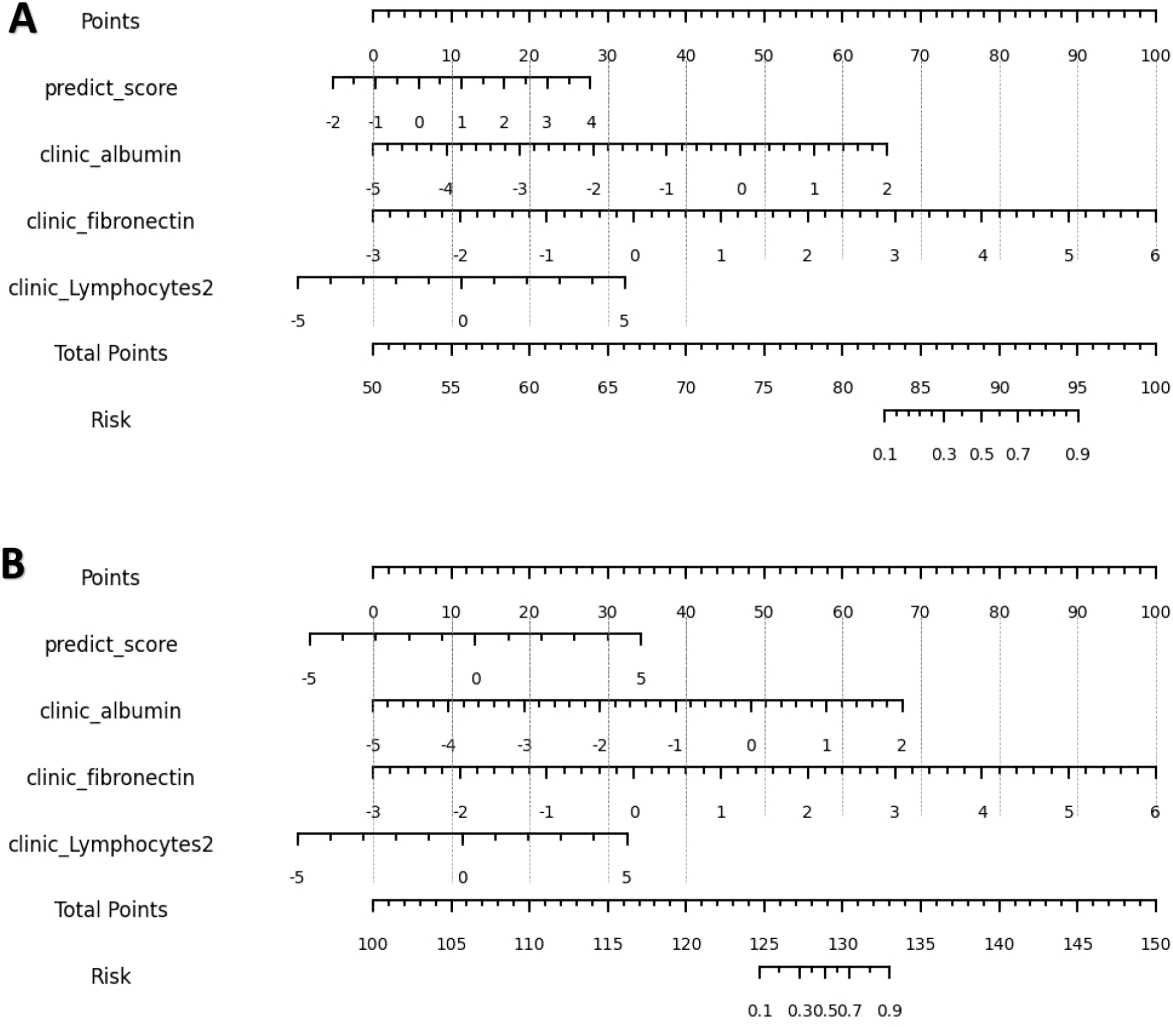
**(A, B)** are nomograms constructed by L-Lab-LR and S-Lab-LR diagnostic models, respectively. L-Lab-LR Rad Score = -1.964×clinic_fibronectin -1.613×clinic_albumin +0.975×predict_score -0.742×clinic_Lymphocytes2 + 0.474. S-Lab-LR Rad Score = -1.942×clinic_fibronectin -1.649×clinic_albumin +0.745×predict_score -0.741×clinic_Lymphocytes2 + 0.393.

The calibration curve (**Figure 7**) showed that the predicted values of the different joint models were in good agreement with the actual values. The cluster heatmap (**Figure 8**) showed significant differences in predict_score, best laboratory data between DM patients and normal controls. It indicates that there are indeed differences in laboratory data and chest CT data between DM patients and normal subjects. By comparing the clustering results, real labels and heat maps, it is helpful to control the quality of the data and intuitively show the difference in the distribution of characteristics between DM patients and normal controls. On the basis of the LR model, Nomogram (**Figure 9**) integrates the predict_score constructed by the optimal image features with laboratory features, and uses the line segment with a certain proportion to draw on the same plane to express the relationship between variables in the diagnostic model.

D2, D3, D4, D5 external validation groups for model validation: (1) The AUCs of L-Lab-LR model were 0.844(95%CI=0.734-0.955), 0.752(95%CI=0.686-0.854), 0.82(95%CI=0.722-0.918) and 0.83 (95% CI = 0.711-0.949). The results of 1000 Bootstrap resampling: average sensitivity= 0.77(95%CI=0.734-0.955). (2) The AUCs of L-Lab-SVM model were 0.846 (95% CI = 0.735-956), 0.754 (95% CI = 0.612-0.868), 0.82 (95% CI = 0.722-0.918) and 0.838 (95% CI = 0.719-0.957). The results of 1000 Bootstrap resampling: average sensitivity=0.78(95%CI=0.747 -0.923). (3) The AUCs of R-Lab-LR models were 0.989 (95% CI = 0.973-1), 0.712 (95% CI = 0.602-0.822), 0.845 (95% CI = 0.732-0.957) and 0.849(95%CI= 0.726-0.972). The results of 1000 Bootstrap resampling: average sensitivity=0.703(95%CI=0.689-0.979). (4) The AUCs of R-Lab-SVM model were 0.971(95%CI= 0.936-1), 0.689(95%CI= 0.577-0.801), 0.852 (95% CI = 0.714-0.967) and 0.804 (95% CI = 0.782-0.926). The results of 1000 Bootstrap resampling: average sensitivity=0.796(95%CI=0.742-0.988).

### Comparison of fasting blood glucose, 2-h plasma glucose with diagnostic model for screening DM

According to the criteria of fasting blood glucose diagnosis of DM (≥7 (mmol/L)), 946 cases were positive and 814 cases were negative in D1 group (false negative rate was about 46.24%). According to the criteria of 2-h plasma glucose (2-h PG) during a 75-g oral glucose tolerance test (OGTT) diagnosis of DM (≥11.1 mmol/L), 1162 cases were positive and 598 cases were negative in D1 group (false negative rate was about 33.98%). Among 1705 non-DM patients, 505 were positive and 1200 were negative (false positive rate was 29.61%). As shown in **Table 3**, for 5138 samples, the number of false negative cases diagnosed by L-Lab-LR_test was 334 (false negative rate was 6.50%), and the number of false positive cases was 258 (false positive rate was 3.56%). For 5138 samples, the number of false negative cases diagnosed by L-Lab-SVM_test was 123 (false negative rate was 2.39%), and the number of false positive cases was 258 (false positive rate was 5.02%). For 5179 samples, the false negative number of S-Lab-LR_test was 164 (false negative rate 3.17%) and the false positive number was 329 (false positive rate 6.35%). For 5270 cases, the false negative number of S-Lab-SVM_test diagnosis was 194 (false negative rate was 3.68%), and the false positive number was 314 (false positive rate was 5.96%).

## Discussion

In this study cohort, the false negative rate of screening patients with diabetes mellitus (DM) based on fasting blood glucose (FBG) physical examination items was as high as 46.24%, which is a very striking figure. In other words, if large-scale screening of DM was carried out based on FBG alone, nearly half of DM patients would be preliminarily judged as having normal fasting glucose (NFG), which would cause a large proportion of patients to delay seeking medical care. As mentioned above, chest CT plain scan is an important method for routine screening of diseases in medical institutions. If the pancreas data of patients scanned by chest CT can be fully utilized and combined with laboratory data, the false negative rate of FBG will be greatly reduced.

In this study, laboratory clinical models and four joint models based on chest CT pancreatic radiomics features and clinical laboratory data were developed and validated for DM screening.

To enhance screening for patients with DM, we identified the optimal laboratory parameters: albumin, fibronectin, and lymphocytes, as well as key imaging metrics, including 2D maximal diameter, 2D mesh surface, 2D minor axis length, and 2D perimeter. As clinical data scores increase—with albumin rising from -5 to 2, interferon from -5 to 6, and lymphocytes from approximately -2.8 to 5—the probability of high diabetes mellitus (DM) risk increases. Similarly, when the integrated predictive score from imaging data increases from -1 to 4 or -3 to 5, (epending on whether the L-Lab-LR or S-Lab-LR is used, the likelihood of high DM risk also increases. This combination of clinical laboratory data and imaging features offers a comprehensive assessment to distinguish high-risk DN patients from those without the condition. The laboratory clinical diagnostic model, serving as the baseline model, initially demonstrated the good diagnostic efficacy of the regression model for DM, with an area under the curve (AUC) of 0.877 (95% CI = 0.867-0.887). Although its specificity reached 0.903, its sensitivity was only 0.75, indicating that it was still prone to missed diagnoses when diagnosing DM. The results indicate that after the image data were combined, the joint models not only exhibited excellent diagnostic performance in the test group of the development center but also showed good diagnostic performance in other validation centers. The S-Lab-LR training model exhibited the highest sensitivity of 0.9 (95% CI = 0.888-0.911), which would greatly reduce the chance of missed diagnoses. Additionally, the L-Lab-SVM training model had the highest specificity of 0.96 (95% CI = 0.952-0.967).

Wang (21) et al, have pointed out that in type 2 diabetes, an imbalance between T helper cell 17 (Th17) and regulatory T cells (Treg) has been observed. In simple terms, Th17 cells act like “accelerators”, promoting inflammation as Treg cells act like “brakes”, suppressing immune responses to maintain tolerance. In conditions of type 2 diabetes and obesity, it is common for the “accelerator” Th17 to be overly active while the “brake” Treg to be inadequately functioning. This imbalance leads to chronic inflammation in tissues and throughout the body, which in turn exacerbates insulin resistance and damage to β -cell function. In type 1 diabetes, the role of lymphocytes is more direct: cytotoxic CD8+ T lymphocytes and other immune cells are activated, specifically recognizing and attacking pancreatic β cells that secrete insulin, resulting in absolute insulin deficiency. This makes lymphocytes and their specific responses the core of the pathogenesis of type 1 diabetes and a hot topic in biomarker research. Fibrinogen (FIB) is a glycoprotein synthesized by the liver and is an important component involved in blood coagulation and hemostasis functions. Previous studies have found that FIB is elevated in inflammatory diseases and is also involved in regulating tissue inflammatory responses (22, 23). In patients with type 2 diabetes, a chronic low-grade inflammatory state stimulates fibrinogen production, leading to an increase in fibrinogen levels in the blood (24). albumin (ALB) plays a crucial role in human plasma. It is the core protein synthesized and secreted by liver cells. It not only provides nutrition and maintains osmotic pressure, but also serves as an acute negative phase reactant of inflammation. Research has found that ALB may accelerate the progression of chronic vascular complications related to diabetes through its antioxidant, anti-inflammatory and anticoagulant effects (25). Fibrinogen and albumin, as two indicators that change in opposite directions, their combination amplifies inflammatory signals in the body.

With the development of radiomics, radiomics features allow the relationship between pancreas and DM to be further explained (11–13). However, previous studies mainly used abdominal CT, which is difficult to achieve large-scale screening in routine physical examination projects. In recent years, Kun (10) et al. conducted a large-scale study using pure clinical data to fully explore the predictive value of clinical data for the risk of DM in the population and showed good predictive efficiency. This study combined laboratory data and imaging data to construct a variety of diagnostic models for early and accurate screening of DM patients. Similar to Kun (10) et al. ‘s study, this study starts from physical examination data and strives to build a diagnostic model that can screen DM patients early and accurately based on physical examination data. In contrast, Kun (10) et al. ‘s study incorporated height, weight, BMI, age and gender into the model construction, while the original intention of this study was to use simple laboratory data and collect the number of DM patients and normal controls in a balanced manner, so as to avoid the situation that the number of normal controls was much larger than that of DM patients.

At the same time, this study combined the plain CT scan data of the physical examination, and made full use of the radiomics features of the pancreas and the laboratory data of the physical examination to construct a diagnostic model. Compared with FBG (false negative rate 46.24%), the false negative rate of the diagnostic model in this study was only 3.17%-6.50%, and the accuracy was as high as 0.899-0.926. It is suggested that if the chest CT scan data and laboratory data are fully used, DM patients can be screened more accurately. Multi-center validation was also performed in this study, and the validation AUCs were > 0.8 for all centers except D3 dataset. The chance of the results from a single center was avoided, which further demonstrates that the diagnostic model constructed in this study is reliable and has the hope to achieve universal promotion.

Several imaging studies have found that fatty pancreas is associated with insulin resistance and β-cell dysfunction (26). These findings by Costabile (26) et al. suggest that pancreatic lipid content may contribute to beta-cell dysfunction and potentially to the subsequent development of type 2 diabetes in susceptible individuals. There are also studies that have found, conversely, that the fat content of the pancreas is not associated with DM or insulin resistance (27). High pancreatic fat content does not necessarily seem to be associated with insulin resistance and β-cell dysfunction. Studies have shown that obesity only acts as a risk factor for fatty pancreas, and obesity increases the risk of fatty pancreas (28). At the same time, a large number of non-obese people suffer from DM (29). Lu (11) et al. proposed that some structural changes reflected by pancreatic radiomics features in DM patients may also be independent of fatty infiltration, and radiomics features may become possible biomarkers for new-onset DM in normal-weight individuals. In the present study, many patients with DM were of normal weight, and the sex ratio was also balanced. Therefore, we manually excluded non-laboratory indicators such as height, weight, and BMI from the physical examination items.

To the best of our knowledge, this is the first study to construct a DM screening model by combining pancreatic features of plain chest CT with laboratory data. Lu (11) et al. constructed three radiomics features based on abdominal CT scans using CT image features extracted from three regions of interest (i.e., the pancreas, liver, and psoas major muscle). By combining radiomics features and other markers, a radiomics nomogram was constructed for screening early diabetes and predicting future diabetes. Jang (13) et al. proposed based on abdominal CT scans that computerized 3D CT texture analysis of the pancreas might be helpful in predicting diabetes. Higher variance, sphericity, GLCM entropy and lower GLCM contrast are important predictive factors for diabetes. The similarity between this study and the previous two is that the target organ studied is the pancreas. The difference lies in that this study was based on chest CT scans. As mentioned earlier, chest CT has become a routine item in current daily physical examinations. Compared with abdominal CT, chest CT is used more frequently in routine physical examinations and has extremely high potential for promotion. This study innovatively adopted the pancreatic texture features in plain chest CT scans and combined them with key laboratory data. Compared with the results of Lu (11) and Jang (13) et al., it has higher AUC, sensitivity and specificity. Different radiomics features can describe or reflect different information. For example, order features can quantitatively describe the distribution of voxels in an image. The characteristics of gray level co-occurrence matrix can reflect the homogeneity and heterogeneity of lesions. The features of gray-scale run-length matrix can reflect the directionality and roughness of image texture (30). The specific condition of this study was to obtain the pancreatic radiomics features from the chest CT scan, so that the diagnostic model could be constructed by routine physical examination, without the need for additional abdominal CT, which ensured the efficient utilization of medical resources.

This study has the following limitations. Firstly, due to the limitation of the software processing platform, it was not possible to process a larger sample in this study. However, in this study, the model was trained and tested based on 3587 cases in D1 center. The pancreatic radiomics features of lung window and soft tissue window of each DM patient and normal control were obtained, which reached tens of thousands of samples, and the data were enriched and reliable. Second, although this study was conducted in multiple centers, CT scanners and related parameters could not be unified. The lower AUC of the D3 validation data may be due to the use of CT scanners from different manufacturers. It is also not possible to test the consistency of CT scanners across different institutions, since it is difficult to obtain CT scans from five institutions for the same patient. Furthermore, the external validation data for D2-D5 is relatively small. However, this study conducted external validation from four new centers, and the validation results were different, indicating that the results are reliable. To achieve the generalization ability of the model, a larger external validation set is needed for verification, which will also become the focus of future research. It needs to be emphasized that the DM group in D1 was significantly older than the non-DM group. Given that age is a well-established, strong risk factor for metabolic diseases, this imbalance represents a potential source of confounding. It is important to clarify that our primary aim was to evaluate the intrinsic predictive capability of the chest CT and laboratory data themselves, independent of age. Consequently, we deliberately constructed our model without including age to test this specific hypothesis. While this choice allows for a clear interpretation of the biomarker’s standalone value, it limits the model’s utility for absolute risk estimation in a clinical setting. Future studies aimed at developing a comprehensive clinical prediction tool should unequivocally incorporate age, along with other established risk factors, to maximize predictive accuracy. In this study, only cross-sectional CT images of the pancreas were selected for analysis, and the whole pancreas was not covered, that is, only 2D analysis was performed. However, NG et al. (31) showed that texture analysis of the maximum cross-sectional area can be used as an alternative to overall texture analysis when performing radiomics analysis. Of course, whether 3D pancreatic texture analysis can further improve the accuracy of diagnostic models is a direction of future research. As the data is only standardized and normalized, there may be data deviations. Moreover, this study only distinguished normal controls from diabetic patients and did not conduct further in-depth research such as the classification of diabetes. Correspondingly, the model constructed in this study should not be applied to predictions in other fields. In this study, a diagnostic model was established by integrating clinical laboratory data and two - dimensional features of plain chest CT scans. Regarding the screening of DM, it not only demonstrates high diagnostic efficacy but also significantly reduces the false - negative rate of traditional methods, which can aid in the early detection and diagnosis of DM in clinical practice. The machine learning models were selected based on their suitability for clinical translation, particularly emphasizing interpretability and robustness to overfitting given the sample size. Logistic Regression was chosen for its ability to provide transparent, quantifiable feature coefficients and probabilistic outputs. Support Vector Machine with a linear kernel was selected for its strong theoretical grounding in maximizing the generalization margin. Both models are well-established in medical informatics for high-dimensional, limited-sample data, providing a strong baseline for evaluating the predictive power of our feature set. This research pertains to the field of machine learning and does not involve in - depth exploration of deep learning and related network models. With the advancement of deep learning, this should become the focal point of future research. The combined diagnostic model constructed in this study was based on clinical laboratory data and the features of chest CT imaging, where the clinical data encompassed invasive examinations. Future research should concentrate on iterating machine learning, integrating deep learning and other technologies to attempt to achieve non - invasive screening of DM.

It should be noted that the four combined models constructed in this study all demonstrated extremely outstanding diagnostic performance (AUC consistently exceeded 0.95 in most cases), indicating that they have a very high ability to distinguish between diabetic patients and non-diabetic patients. This means that the method of this study can identify patients with extremely high accuracy, especially those who may not be diagnosed by traditional methods alone. Therefore, the joint model strategy proposed in this study is not intended to replace the existing methods, but to provide a powerful supplement to them. It is like adding a “highly sensitive” safety net to the existing screening system, which can greatly reduce the rate of clinical missed diagnosis and ensure that more high-risk groups are detected and intervened in time at the early stage of the disease. This strategy that combines the macroscopic morphological information of radiomics with the microscopic biochemical indicators of the laboratory has opened up a new dimension for the early diagnosis of diabetes and demonstrated great potential and value for clinical transformation. The results of this study indicate that the secondary utilization of conventional chest CT scans for diabetes detection is anticipated to enhance public health and cost-effectiveness: This method can make secondary use of the chest CT pancreatic data from the physical examination items of the examinee, which not only greatly reduces the false negative rate of DM screening, but also fully utilizes medical resources and reduces medical costs.

## Conclusion

In this study, the diagnostic models established based on a large-scale sample of physical chest CT pancreatic radiomics features and laboratory data exhibit high accuracy in diagnosing patients with DM. These models have significantly decreased the false-negative rate of traditional methods and are both feasible and reliable for the preliminary screening of DM patients. This approach has been demonstrated to be superior in identifying individuals with DM, which will contribute to the early diagnosis of DM patients who may have been missed.

## Data Availability

The raw data supporting the conclusions of this article will be made available by the authors, without undue reservation.

## References

[1] Green JB Everett BM Ghosh A Younes N Krause-Steinrauf H Barzilay J . Cardiovascular outcomes in GRADE (Glycemia reduction approaches in type 2 diabetes: A comparative effectiveness study). Circulation. (2024) 149:993–1003. doi: 10.1161/CIRCULATIONAHA.123.066604, PMID: 38344820 PMC10978227

[2] Crider K Williams J Qi YP Gutman J Yeung L Mai C . Folic acid supplementation and malaria susceptibility and severity among people taking antifolate antimalarial drugs in endemic areas. Cochrane Database Syst Rev. (2022) 2:CD014217. doi: 10.1002/14651858.CD014217, PMID: 36321557 PMC8805585

[3] Federation ID . IDF diabetes atlas, 10th ed. Liuzhou (2021)

[4] Redondo MJ Warnock MV Libman IM Bocchino LE Cuthbertson D Geyer S . TCF7L2 genetic variants do not influence insulin sensitivity or secretion indices in autoantibody-positive individuals at risk for type 1 diabetes. Diabetes Care. (2021) 44:2039–44. doi: 10.2337/dc21-0531, PMID: 34326068 PMC8740915

[5] Nanditha A Thomson H Susairaj P Srivanichakorn W Oliver N Godsland IF . A pragmatic and scalable strategy using mobile technology to promote sustained lifestyle changes to prevent type 2 diabetes in India and the UK: a randomised controlled trial. Diabetologia. (2020) 63:486–96. doi: 10.1007/s00125-019-05061-y, PMID: 31919539 PMC6997257

[6] Watkins DA Ali MK . Measuring the global burden of diabetes: implications for health policy, practice, and research. Lancet. (2023) 402:163–5. doi: 10.1016/S0140-6736(23)01287-4, PMID: 37356449

[7] Nazarzadeh M Bidel Z Canoy D Copland E Bennett DA Dehghan A . Blood pressure-lowering treatment for prevention of major cardiovascular diseases in people with and without type 2 diabetes: an individual participant-level data meta-analysis. Lancet Diabetes Endocrinol. (2022) 10:645–54. doi: 10.1016/S2213-8587(22)00172-3, PMID: 35878651 PMC9622419

[8] Nomura A Noguchi M Kometani M Furukawa K Yoneda T . Artificial intelligence in current diabetes management and prediction. Curr Diabetes Rep. (2021) 21:61. doi: 10.1007/s11892-021-01423-2, PMID: 34902070 PMC8668843

[9] Makroum MA Adda M Bouzouane A Ibrahim H . Machine learning and smart devices for diabetes management: systematic review. Sensors (Basel). (2022) 22:1843. doi: 10.3390/s22051843, PMID: 35270989 PMC8915068

[10] Lv K Cui C Fan R Zha X Wang P Zhang J . Detection of diabetic patients in people with normal fasting glucose using machine learning. BMC Med. (2023) 21:342. doi: 10.1186/s12916-023-03045-9, PMID: 37674168 PMC10483877

[11] Lu CQ Wang YC Meng XP Zhao HT Zeng CH Xu W . Diabetes risk assessment with imaging: a radiomics study of abdominal CT. Eur Radiol. (2019) 29:2233–42. doi: 10.1007/s00330-018-5865-5, PMID: 30523453

[12] ZZeng N Wang Y Cheng Y Huang Z Song B . Imaging evaluation of the pancreas in diabetic patients. Abdom Radiol (NY). (2022) 47:715–26. doi: 10.1007/s00261-021-03340-0, PMID: 34786594

[13] Jang S Kim JH Choi SY Park SJ Han JK . Application of computerized 3D-CT texture analysis of pancreas for the assessment of patients with diabetes. PloS One. (2020) 15:e0227492. doi: 10.1371/journal.pone.0227492, PMID: 31929591 PMC6957148

[14] Canovi S Besutti G Bonelli E Iotti V Ottone M Albertazzi L . The association between clinical laboratory data and chest CT findings explains disease severity in a large Italian cohort of COVID-19 patients. BMC Infect Dis. (2021) 21:157. doi: 10.1186/s12879-021-05855-9, PMID: 33557778 PMC7868898

[15] Salvatore C Roberta F Angela L Cesare P Alfredo C Giuliano G . Clinical and laboratory data, radiological structured report findings and quantitative evaluation of lung involvement on baseline chest CT in COVID-19 patients to predict prognosis. Radiol Med. (2021) 126:29–39. doi: 10.1007/s11547-020-01293-w, PMID: 33047295 PMC7549421

[16] Li K Wu J Wu F Guo D Chen L Fang Z . The clinical and chest CT features associated with severe and critical COVID-19 pneumonia. Invest Radiol. (2020) 55:327–31. doi: 10.1097/RLI.0000000000000672, PMID: 32118615 PMC7147273

[17] Sohn JH Fields BKK . Radiomics and deep learning to predict pulmonary nodule metastasis at CT. Radiology. (2024) 311:e233356. doi: 10.1148/radiol.233356, PMID: 38591975

[18] Zabor EC Reddy CA Tendulkar RD Patil S . Logistic regression in clinical studies. Int J Radiat Oncol Biol Phys. (2022) 112:271–7. doi: 10.1016/j.ijrobp.2021.08.007, PMID: 34416341

[19] Wang H Shao Y Zhou S Zhang C Xiu N . Support vector machine classifier via L0/1 soft-margin loss. IEEE Trans Pattern Anal Mach Intell. (2022) 44:7253–65. doi: 10.1109/TPAMI.2021.3092177, PMID: 34166185

[20] Balch JA Ruppert MM Guan Z Buchanan TR Abbott KL Shickel B . Risk-specific training cohorts to address class imbalance in surgical risk prediction. JAMA Surg. (2024) 159:1424–31. doi: 10.1001/jamasurg.2024.4299, PMID: 39382865 PMC11465118

[21] Wang M Chen F Wang J Zeng Z Yang Q Shao S . Th17 and Treg lymphocytes in obesity and Type 2 diabetic patients. Clin Immunol. (2018) 197:77–85. doi: 10.1016/j.clim.2018.09.005, PMID: 30218707

[22] Hsieh JY Smith TD Meli VS Tran TN Botvinick EL Liu WF . Differential regulation of macrophage inflammatory activation by fibrin and fibrinogen. Acta Biomater. (2017) 47:14–24. doi: 10.1016/j.actbio.2016.09.024, PMID: 27662809 PMC5426227

[23] Luyendyk JP Schoenecker JG Flick MJ . The multifaceted role of fibrinogen in tissue injury and inflammation. Blood. (2019) 133:511–20. doi: 10.1182/blood-2018-07-818211, PMID: 30523120 PMC6367649

[24] Yang SH Du Y Zhang Y Li XL Li S Xu RX . Serum fibrinogen and cardiovascular events in Chinese patients with type 2 diabetes and stable coronary artery disease: a prospective observational study. BMJ Open. (2017) 7:e015041. doi: 10.1136/bmjopen-2016-015041, PMID: 28601829 PMC5734258

[25] Zhu Y Cai X Liu Y Hu M Zhou L Liu W . Serum Albumin, but not Bilirubin, is Associated with Diabetic Chronic Vascular Complications in a Chinese Type 2 Diabetic Population. Sci Rep. (2019) 9:12086. doi: 10.1038/s41598-019-48486-6, PMID: 31427625 PMC6700065

[26] Costabile G Salamone D Della Pepa G Testa R Vitale M Brancato V . ApoC-III and ectopic fat accumulation in individuals with type 2 diabetes: an exploratory analysis from the MEDEA randomised controlled study. Diabetologia. (2025) 68:2036–41. doi: 10.1007/s00125-025-06464-w, PMID: 40471240

[27] Wagner R Jaghutriz BA Gerst F Barroso Oquendo M Machann J Schick F . Pancreatic steatosis associates with impaired insulin secretion in genetically predisposed individuals. J Clin Endocrinol Metab. (2020) 105:3518–25. doi: 10.1210/clinem/dgaa435, PMID: 32725157 PMC7497818

[28] Szczerbinski L Florez JC . Precision medicine of obesity as an integral part of type 2 diabetes management - past, present, and future. Lancet Diabetes Endocrinol. (2023) 11:861–78. doi: 10.1016/S2213-8587(23)00232-2, PMID: 37804854

[29] Liu Y Kimita W Shamaitijiang X Skudder-Hill L Sequeira-Bisson IR Petrov MS . Intra-pancreatic fat is associated with continuous glucose monitoring metrics. Diabetes Obes Metab. (2024) 26:2359–67. doi: 10.1111/dom.15550, PMID: 38528823

[30] Qi YJ Su GH You C Zhang X Xiao Y Jiang YZ . Radiomics in breast cancer: Current advances and future directions. Cell Rep Med. (2024) 5:101719. doi: 10.1016/j.xcrm.2024.101719, PMID: 39293402 PMC11528234

[31] Ng F Kozarski R Ganeshan B Goh V . Assessment of tumor heterogeneity by CT texture analysis: can the largest cross-sectional area be used as an alternative to whole tumor analysis? Eur J Radiol. (2013) 82:342–8. doi: 10.1016/j.ejrad.2012.10.023, PMID: 23194641

